# Supplementary material for: Disability among Women and Men Who Married in Childhood: Evidence from Cross-Sectional Nationally Representative Surveys Undertaken in 37 Low- and Middle-Income Countries
Source: Int J Environ Res Public Health. 2022 Dec 21;20(1):88. doi: 10.3390/ijerph20010088 (PMC9819517; doi:10.3390/ijerph20010088)
Supplement: Supplementary file 1 [file ijerph-20-00088-s001.zip › ijerph-2055383 - supplementary.pdf]

International Journal of
Environmental Research and Public Health

MDPI

Supplementary Table S1. Prevalence of Disability Stratified by Participant Age, Household Wealth, Highest Level of Education and Country Economic Classification Group.

	Women		Men	
	**Prevalence of disability**	**Unadjusted prevalence rate ratio**	**Prevalence of disability**	**Unadjusted prevalence rate ratio**
		Age		
under 22<br>Nwomen = 67,884, Nmen=16,798	9.0%<br>(8.3-9.9)	1.0 (reference)	8.0%<br>(7.0-9.0)	1.0 (reference)
22-25<br>Nwomen = 65,699, Nmen=13,585	8.7%<br>(8.0-9.4)	1.02<br>(0.99-1.06)	7.6%<br>(6.7-8.6)	0.92*<br>(0.85-1.00)
26-29<br>Nwomen = 60,907, Nmen=12,409	10.2%<br>(9.5-11.0)	1.17***<br>(1.13-1.21)	8.1%<br>(7.3-9.0)	0.99 (0.91-1.12)
30-33<br>Nwomen = 58,328, Nmen=12,518	11.6%<br>(10.7-12.6)	1.37***<br>(1.32-1.42)	8.2%<br>(7.3-9.3)	1..03<br>(0.95-1.12)
34-37<br>Nwomen = 52,918, Nmen=11,541	14.7%<br>(13.6-15.8)	1.71***<br>(1.65-1.77)	9.4%<br>(8.3-10.7)	1.20***<br>(1.10-1.30)
38-41<br>Nwomen = 45,957, Nmen=10,341	19.4%<br>(18.2-20.7)	2.27***<br>(2.19-2.35)	11.4%<br>(10.1-12.8)	1.43***<br>(1.32-1.55)
42-45<br>Nwomen = 39,378, Nmen=9,251	25.3%<br>(24.9-26.7)	3.01***<br>(2.91-3.11)	14.5%<br>12.7-16.4	1.88***<br>(1.74-2.02)
46-49<br>Nwomen = 32,094, Nmen=7,825	31.4%<br>(29.9-33.2)	3.76***<br>(3.64-3.88)	19.8%<br>(17.7-22.1)	2.53***<br>(2.34-2.72)
		Within Country Household Wealth Quintile		
Richest<br>Nwomen = 78,424, Nmen=17,787	12.2%<br>(11.1-13.4)	1.0 (reference)	7.8%<br>(6.9-8.8)	1.0 (reference)
4<br>Nwomen = 81,897, Nmen=18,210	14.0%<br>(13.0-15.1)	1.10***<br>(1.07-1.13)	9.6%<br>(8.6-10.7)	1.11**<br>(1.04-1.19)
3<br>Nwomen = 84,028, Nmen=18,436	14.9%<br>(13.8-16.0)	1.17***<br>(1.14-1.20)	10.9%<br>(9.8-12.2)	1.24***<br>(1.16-1.33)
2<br>Nwomen = 86,1879,<br>Nmen=19,735	15.8%<br>(14.6-17.1)	1.22***<br>(1.19-1.25)	11.7%<br>(10.3-13.2)	1.32***<br>(1.24-1.42)
Poorest<br>Nwomen = 92,637, Nmen=21,243	17.1%<br>(15.7-18.6)	1.33***<br>(1.29-1.36)	13.7%<br>(12.3-15.3)	1.52***<br>(1.42-1.62)
		Highest Level of Education		
Secondary<br>Nwomen = 235,886,<br>Nmen=59,673	12.2%<br>(11.4-13.0)	1.0 (reference)	9.6%<br>(8.8-10.6)	1.0 (reference)
Primary<br>Nwomen = 110,858,<br>Nmen=23,444	18.6%<br>(17.4-19.9)	1.57***<br>(1.53-1.60)	12.6%<br>(11.7-13.5)	1.57***<br>(1.53-1.60)
Pre-primary/None<br>Nwomen = 76,370, Nmen=12,247	17.5%<br>(14.9-20.5)	1.74***<br>(1.70-1.78)	11.6%<br>(8.9-15.0)	1.71***<br>(1.60-1.83)
		Country Economic Classification Group		
Upper-middle income<br>Nwomen = 105,418,<br>Nmen=16,451	15.0%<br>(13.9-16.1)	n/a	9.6%<br>(8.8-10.4)	n/a
Lower-middle income	14.9%<br>(13.6-16.3)	n/a	12.8%<br>(11.6-14.0))	n/a

Nwomen = 191,938, Nmen=38,177				
Low income Nwomen = 125,809, Nmen=40,783	14.1% (11.9-16.6)	n/a	8.8% (7.3-10.6)	n/a

Note: * p<0.05, ** p<0.01, *** p<0.001.

Supplementary Table S2. Prevalence of Child Marriage Stratified by Participant Age, Household Wealth, Highest Level of Education and Country Economic Classification Group.

	**Child Marriage Under 18**		**Child Marriage Under 16**	
**Women**	**Prevalence of disability**	**Unadjusted prevalence rate ratio**	**Prevalence of disability**	**Unadjusted prevalence rate ratio**
		Age		
under 22 N=67,878	28.6% (27.6-29.5)	1.0 (reference)	13.3% (12.8-13.7)	1.0 (reference)
22-25 N=65,696	30.9% (29.5-32.4)	1.09*** (1.07-1.11)	14.9% (14.4-15.5)	1.14*** (1.11-1.18)
26-29 N=60,906	31.0% (29.6-32.4)	1.10*** (1.08-1.13)	15.6% (15.1-16.2)	1.21*** (1.18-1.25)
30-33 N=58,327	32.3% (30.8-33.8)	1.15*** (1.13-1.17)	17.4% (16.6-18.2)	1.36*** (1.32-1.39)
34-37 N=52,916	32.8% (31.0-34.6)	1.17*** (1.15-1.20)	16.5% (15.6-17.4)	1.29*** (1.26-1.33)
38-41 N=45,954	31.4% (29.7-33.0)	1.16*** (1.13-1.18)	16.5% (15.6-17.5)	1.33*** (1.30-1.38)
42-45 N=39,375	30.4% (28.7-32.2)	1.19*** (1.16-1.22)	15.5% (14.4-16.6)	1.36*** (1.32-1.40)
46-49 N=32,093	28.4% (26.5-30.4)	1.16*** (1.13-1.18)	14.3% (13.0-15.6)	1.31*** (1.27-1.36)
		Within Country Household Wealth Quintile		
Richest N=78,422	20.8% (19.5-22.2)	1.0 (reference)	9.8% (9.2-10.4)	1.0 (reference)
4 N=81,894	29.0% (27.7-30.4)	1.38*** (1.35-1.41)	14.1% (13.4-14.8)	1.44*** (1.40-1.48)
3 N=84,024	32.9% (31.6-34.4)	1.56*** (1.53-1.59)	16.7% (16.0-17.5)	1.68*** (1.63-1.72)
2 N=86,174	36.0% (34.4-37.6)	1.69*** (1.66-1.72)	18.6% (17.8-19.5)	1.85*** (1.80-1.90)
Poorest N=92,631	37.4% (35.6-39.2)	1.77*** (1.74-1.80)	19.4% (18.6-20.3)	1.95*** (1.91-2.01)
		Highest Level of Education		
Secondary N=235,876	19.9% (19.0-20.8)	1.0 (reference)	8.2% (7.7-8.7)	1.0 (reference)
Primary N=110,850	44.5% (42.7-46.4)	1.88*** (1.85-1.91)	23.1% (22.2-24.1)	2.29*** (2.25-2.34)
Pre-primary/None N=76,368	48.7% (46.7-50.7)	1.92*** (1.89-1.95)	29.8% (29.0-30.7)	2.49*** (2.44-2.55)
		Country Economic Classification Group		
Upper-middle income N=105,411	22.2% (21.1-23.3)	n/a	9.7% (9.2-10.2)	n/a

Lower-middle income N=191,926	30.4% (27.0-34.1)	n/a	15.1% (13.1-17.4)	n/a
Low income N=125,808	39.0% (37.8-40.2)	n/a	21.2% (20.7-21.7)	n/a
Men				
Age				
under 22 N=16,639	5.3% (4.6-6.2)	1.0 (reference)	1.7% (1.3-2.3)	1.0 (reference)
22-25 N=13,463	7.5% (6.8-8.1)	1.34*** (1.23-1.46)	3.0% (2.6-3.5)	1.58*** (1.37-1.82)
26-29 N=12,343	8.0% (7.4-8.7)	1.51*** (1.39-1.65)	3.4% (2.9-4.0)	2.08*** (1.80-2.39)
30-33 N=12,468	8.9% (8.1-9.7)	1.73*** (1.59-1.88)	4.4% (3.8-5.0)	2.65*** (2.32-3.03)
34-37 N=11,509	9.4% (8.6-10.2)	1.72*** (1.58-1.88)	3.9% (3.1-4.8)	2.19*** (1.91-2.52)
38-41 N=10,309	8.2% (7.1-9.6)	1.51*** (1.38-1.65)	3.7% (2.9-4.8)	2.17*** (1.88-2.51)
42-45 N=9.221	7.9% (7.0-8.8)	1.50*** (1.36-1.64)	3.7% (3.0-4.5)	2.18*** (1.88-2.52)
46-49 N=7,795	8.1% (7.2-9.0)	1.58*** (1.43-1.74)	3.7% (2.9-4.6)	2.30*** (1.97-2.69)
Within Country Household Wealth Quintile				
Richest N=17,689	4.6% (4.2-5.0)	1.0 (reference)	2.0% (1.8-2.3)	1.0 (reference)
4 N=18,119	7.0% (6.4-7.6)	1.59*** (1.45-1.73)	2.8% (2.4-3.2)	1.51*** (1.32-1.72)
3 N=18,329	8.6% (7.9-9.3)	1.87*** (1.72-2.03)	3.6% (2.9-4.3)	1.90*** (1.67-2.16)
2 N=19,628	9.0% (8.0-10.1)	2.05*** (1.89-2.22)	3.9% (3.2-4.9)	2.14*** (1.89-2.42)
Poorest N=21,124	10.8% (9.5-12.2)	2.54*** (2.34-2.74)	4.7% (3.6-6.1)	2.66*** (2.36-3.00)
Highest Level of Education				
Secondary N=59,222	5.6% (5.3-6.0)	1.0 (reference)	2.2% (2.0-2.4)	1.0 (reference)
Primary N=23,389	11.4% (10.6-12.3))	1.83*** (1.74-1.93)	4.8% (4.4-5.2)	1.92*** (1.76-2.08)
Pre-primary/None N=12,232	13.1% (10.2-16.6)	2.05*** (1.92-2.19)	7.2% (4.5-11.5)	2.55*** (2.32-2.81)
Country Economic Classification Group				
Upper-middle income N=15,977	5.3% (3.7-7.5)	n/a	1.8% (1.3-2.6)	n/a
Lower-middle income N=38,159	7.6% (6.5-8.9)	n/a	2.8% (2.3-3.4)	n/a
Low income N=40,753	9.1% (8.2-10.1)	n/a	4.5% (3.5-5.9)	n/a

Note: *** p<0.001.

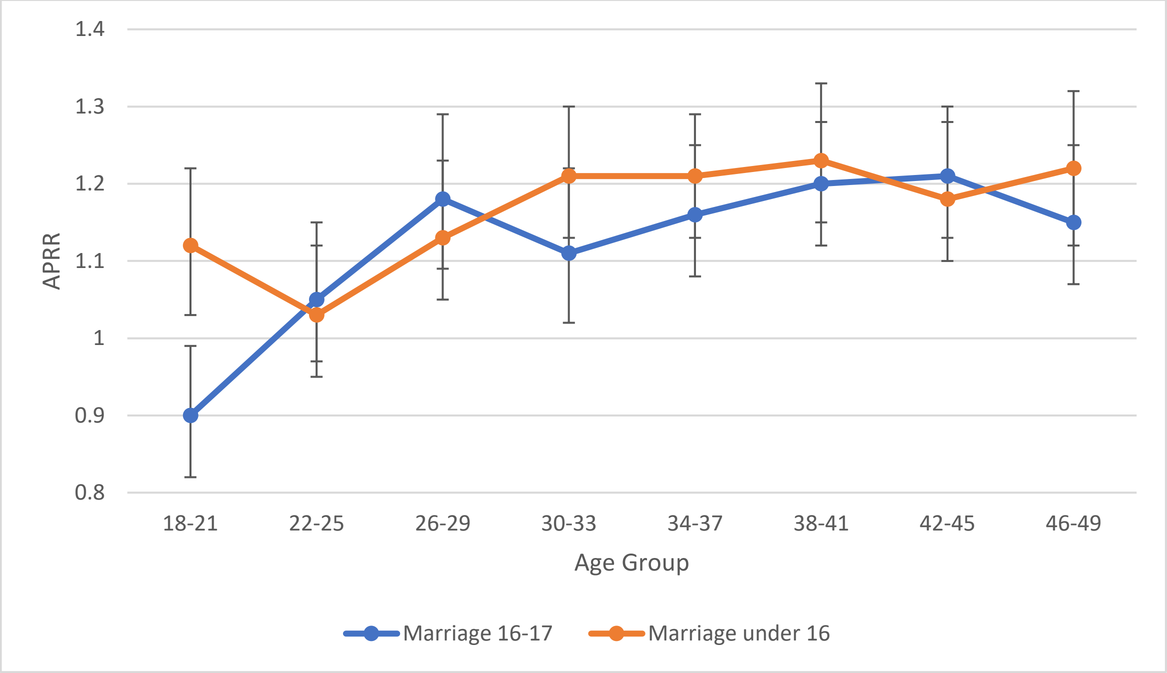

Supplementary Figure S1. Prevalence rate ratios with 95% CIs (adjusted for within age group variation in age) for women with less severe disabilities having been exposed to child marriage at age 16-17 and under 16 by age group.

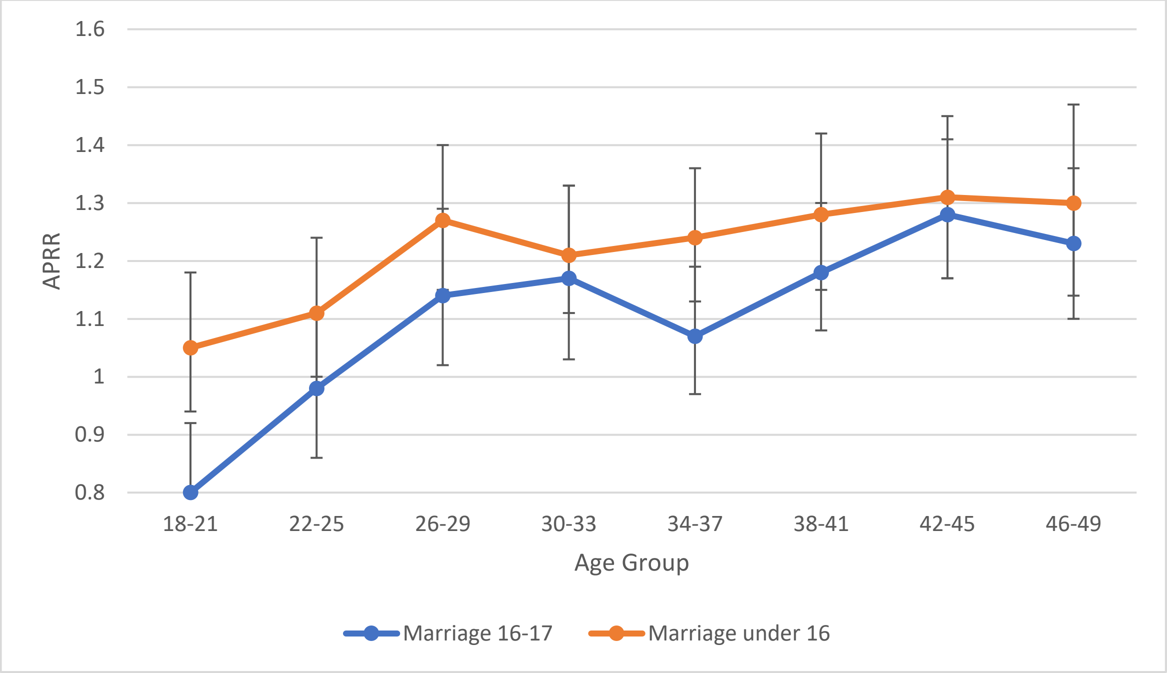

Supplementary Figure S2. Prevalence rate ratios with 95% CIs (adjusted for within age group variation in age) for women with more severe disabilities having been exposed to child marriage at age 16-17 and under 16 by age group.

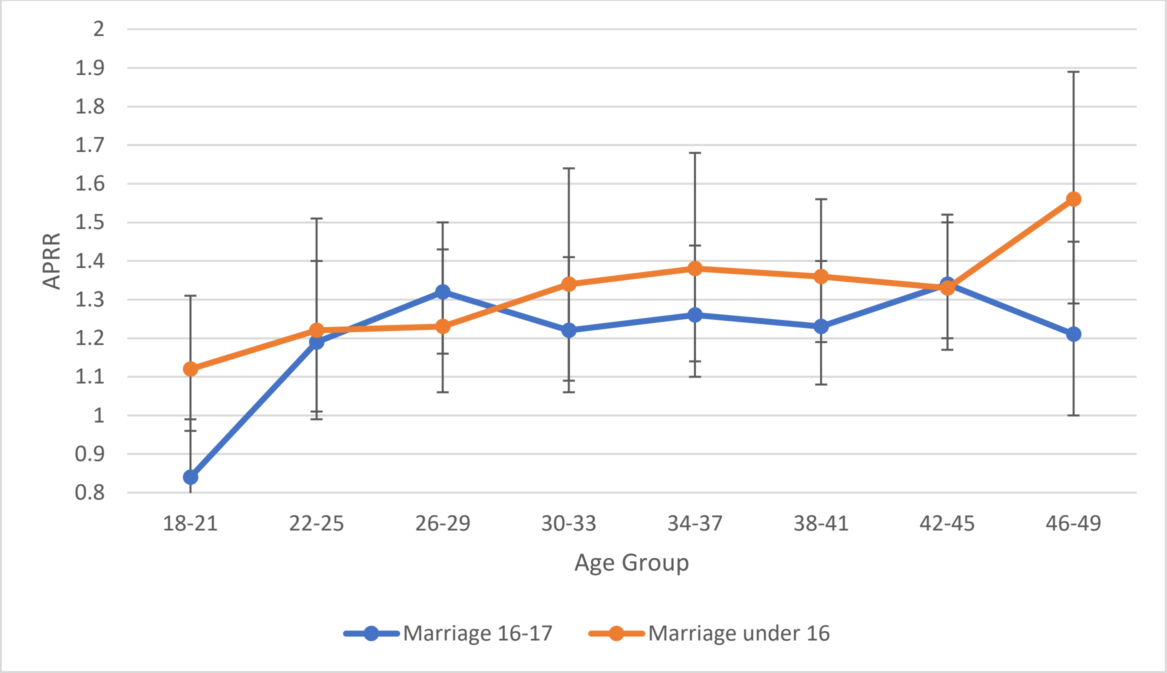

Supplementary Figure S3. Prevalence rate ratios with 95% CIs (adjusted for within age group variation in age) for women with disabilities in Upper-Middle Income countries having been exposed to child marriage at age 16-17 and under 16 by age group.

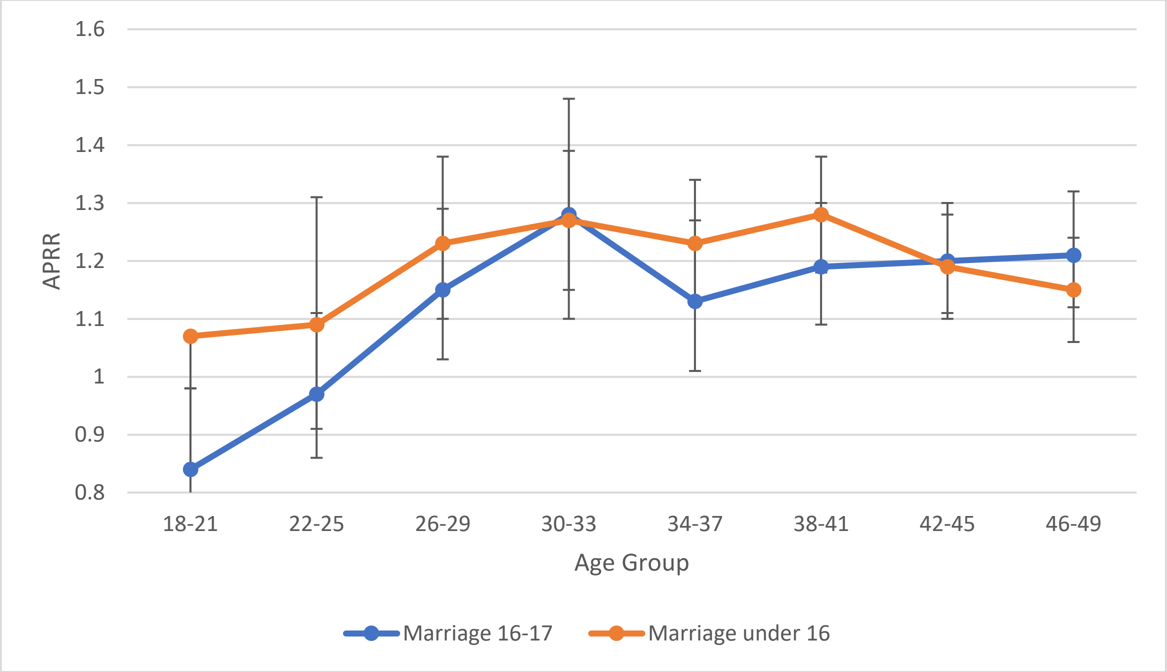

Supplementary Figure S4. Prevalence rate ratios with 95% CIs (adjusted for within age group variation in age) for women with disabilities in Lower-Middle Income countries having been exposed to child marriage at age 16-17 and under 16 by age group.

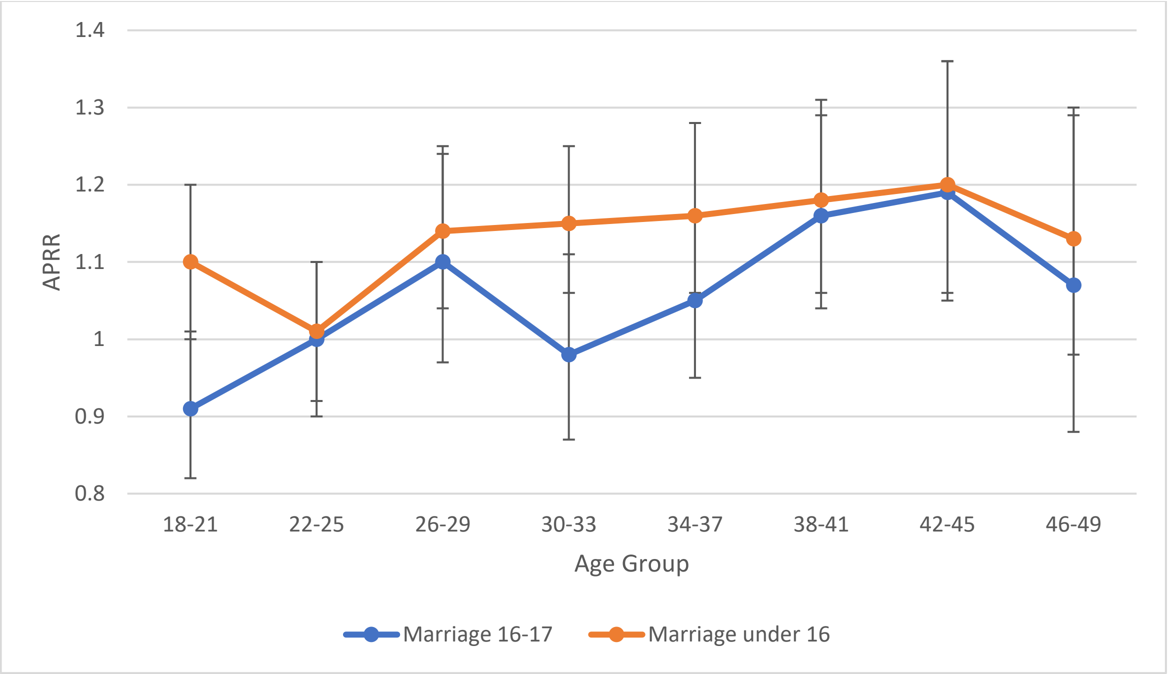

Supplementary Figure S5. Prevalence rate ratios with 95% CIs (adjusted for within age group variation in age) for women with disabilities in Low Income countries having been exposed to child marriage at age 16-17 and under 16 by age group.
